# Ultrasound guided lipofilling as a suggested method for implant salvage in implant based breast reconstruction: A case report

**DOI:** 10.1016/j.ijscr.2019.02.050

**Published:** 2019-03-22

**Authors:** Tarek Hashem, Amira Radwan, Maged Yousry

**Affiliations:** a Breast Surgery Unit, National Cancer Institute, Cairo University, Egypt; b Radiology Department, National Cancer Institute, Cairo University, Egypt

**Keywords:** Implant exposure, Lipofilling, Breast reconstruction, Breast implants, Case report

## Abstract

**Background:**

Implant based reconstruction is one of the well-established methods to restore shape and volume after total mastectomy. Exposure of an implant is a serious complication that could lead to failure of the whole process of breast reconstruction. There are no uniform guidelines on the management of such complication. Several methods have been described including antibiotic irrigation, implant replacement and flap coverage. This case report describes the use of ultrasound guided lipofilling for implant salvage in case of an exposed non-infected implant.

**Case presentation:**

A 29-year-old patient with T3N1 post neoadjuvant chemotherapy breast cancer underwent skin sparing mastectomy and immediate subpectoral implant based reconstruction. She presented a week post hospital discharge with an exposed non infected silicone implant.

Antibiotic irrigation and secondary sutures were done with satisfactory wound healing within eight days. However, ultrasound examination revealed a sizeable area where the implant was in direct contact with the skin.

Utrasound guided lipofilling was done in the same setting as contralateral mastopexy to achieve symmetry. The patient had a smooth postoperative course. She was referred to radiation therapy department where she received adjuvant chest wall irradiation according to her pathological result.

Follow up after radiation therapy by clinical and ultrasound examination revealed no implant related complications.

**Conclusion:**

Lipofilling is a very useful tool that could help in cases of an uninfected exposed implant. It could also be used primarily as an adjunct procedure to implant based reconstruction to optimize the outcome.

## Highlights

• A 27-year-old case of breast cancer underwent skin sparing mastectomy and implant reconstruction.
• The patient presented with an exposed but not infected implant during her follow up.
• After antibiotic irrigation and secondary suture, ultrasound guided lipofilling was performed for implant coverage.
• The patient had a smooth course and received radiotherapy with no other recorded implant - related complications.

## 1 Introduction

Lipofilling is one of the relatively new tools introduced to the field of breast reconstruction. The use of fat to restore a breast mound has its origins in historical reports since 1895 [1].

Over the past two decades, several publications have reported the outcome of large series of patients who underwent lipofilling for partial or total breast reconstruction [2]. The role of fat grafting in breast reconstruction is multifaceted [3].

Implant based breast reconstruction is one of the widely applied techniques to restore shape and volume after mastectomy. In order to decrease implant related complications there should be adequate soft tissue coverage of an implant. Exposure of an implant could be a very disappointing complication to both surgeon and patient. Many cases end up losing their implant with associated financial and emotional burdens.

Implant exposure could be caused by infection, trauma or deficient soft tissue coverage [4].

There are no uniform guidelines on the best practice in cases of implant exposure. Most authors recommend implant removal and immediate or delayed replacement by a new prosthesis. Few reports have described salvage of exposed breast implants in general [5].

This case report demonstrates how lipofilling can aid in salvage of an exposed implant. It has been reported in line with the SCARE criteria [6].

## 2 Case presentation

**Fig. 1:**
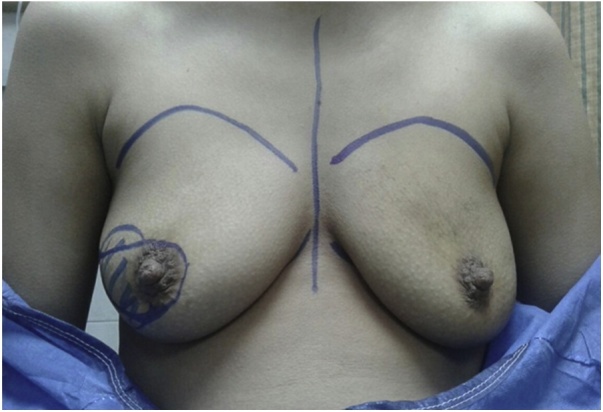
Preoperative picture.

A 29-year-old patient who presented initially with T3 N1 right breast cancer to the breast unit of the National Cancer Institute of Cairo University. The patient received 8 cycles of neoadjuvant chemotherapy with moderate clinical response. A decision was taken for mastectomy and reconstruction. The patient had a BMI of 22.4 kg/m². Her breasts were medium sized (cup 34 B) with second degree ptosis (Fig. 1).

The reconstructive options were discussed with the patient. She expressed a wish for implant based reconstruction preferably with no autologous flaps.

### 2.1 Operative technique

Through a transverse tear drop incision skin sparing mastectomy was completed. A subpectoral pocket was fashioned using the dual plane technique at the inferomedial aspect of pectoralis major muscle. This allowed expansion of the pocket at the level of the inframammary fold.

**Fig. 2:**
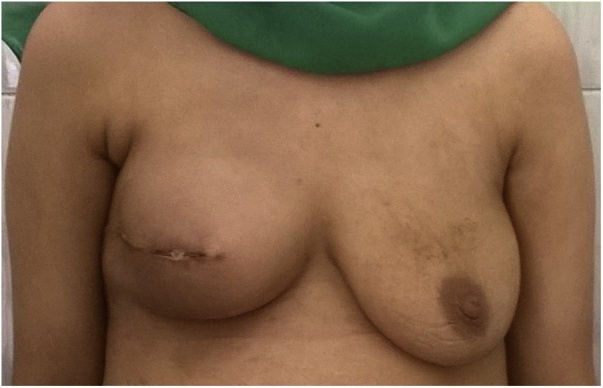
Postoperative picture.

A 275 cc anatomical silicone implant was placed in the pocket to achieve the planned breast mound (Fig. 2).

The patient was discharged 48 h after an uneventful postoperative course. An appointment was scheduled one week after discharge in the outpatient clinic where the drains were removed.

**Fig. 3:**
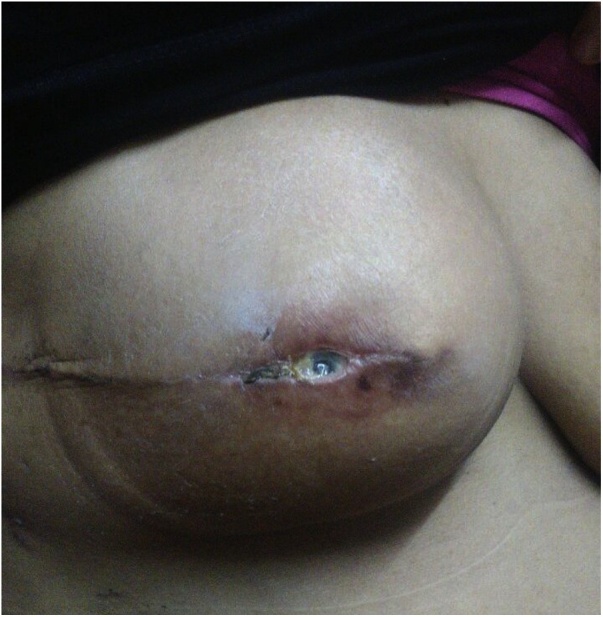
Exposed implant.

One week later the patient presented with implant exposure through the central part of the wound. The implant was exposed but not infected (Fig. 3).

**Fig. 4:**
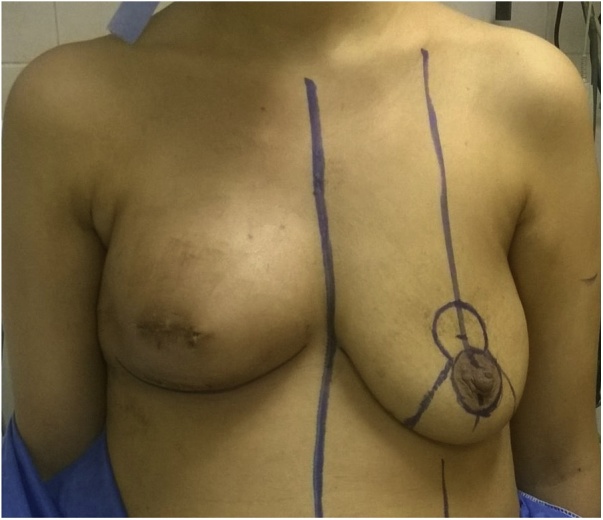
After antibiotic irrigation and secondary sutures.

The patient was scheduled for surgery next morning where copious antibiotic irrigation and secondary sutures were done. The wound showed proper complete healing within eight days and there were no signs of infection (Fig. 4).

Ultrasonographic examination of the reconstructed breast revealed an area of about 3 × 5 cm in direct relation to the scar, where the implant was completely subcutaneous. The patient was planned for lipofilling and contralateral mastopexy.

Planning of donor sites for fat harvest was done preoperatively and according to the patient preference from: lower abdominal area, flanks and trochanteric areas.

### 2.2 Fat harvesting technique

After tumescent fluid infiltration, harvesting of fat using 3,4 mm rounded tip spiral pores cannulas was done using vacuum assisted device into sterile containers.

### 2.3 Fat processing

Harvested fat was repeatedly washed using antibiotic based saline to get rid of blood and tumescent fluid, then transported into centrifuge tubes where one spin for one minute and with 3000 RBM was done. The pure fat was transferred into multiple syringes ready to be injected.

### 2.4 Technique of injection

**Fig. 5:**
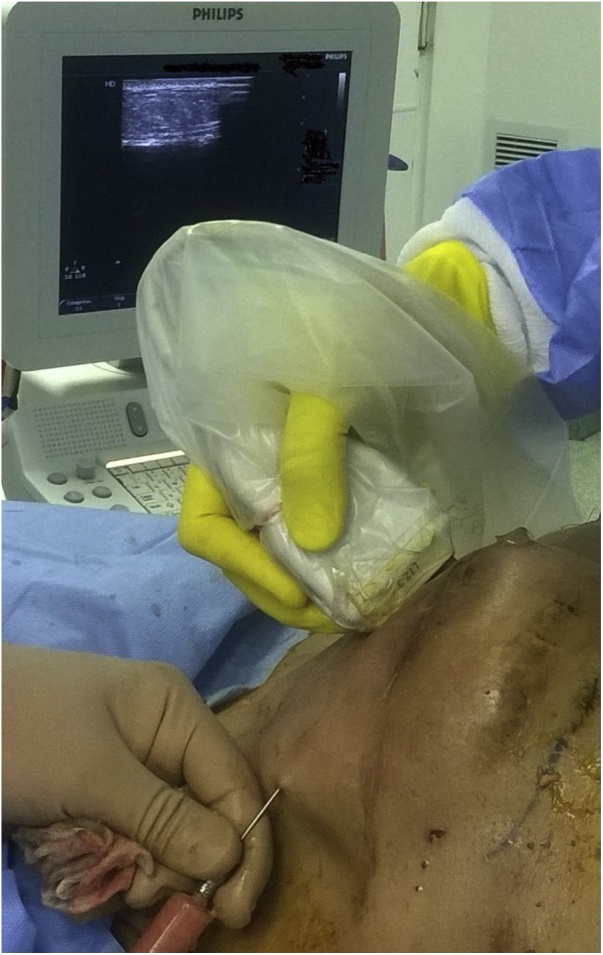
Ultrasound guided lipofilling.

Using single pore curved 2 mm lipofilling needle injection of small amount of fat was done under continuous U/S monitoring to create a space separating the implant and overlying muscle from the skin, then using 3 mm spiral pores cannula repeated fat injection was meticulously done in different directions with average 5–7 cm^3^ of fat per line. Coverage of the whole implant by an average thickness 2.5–3 cm layer of fat graft was achieved under ultrasound guidance (Fig. 5). Filling of the contra-lateral upper medial quadrant was done for adequate symmetrization of both breast. An overall 180–200 cc of harvested fat was injected.

**Fig. 6:**
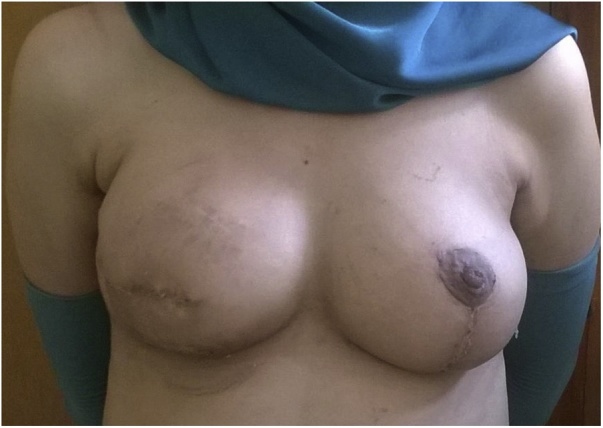
Final post-operative result.

The patient had an uncomplicated postoperative course and was discharged next day. She was reviewed one week later in the outpatient clinic and had clean completely healed wounds (Fig. 6).

The postoperative pathology report revealed a 4.5 × 5.3 cm invasive duct carcinoma with moderate therapy effect and 3/15 positive axillary nodes. Accordingly, the patient was referred to the radiation therapy department.

Chest wall irradiation was administered in a total dose of 50.4 Gy in 28 fractions.

**Fig. 7:**
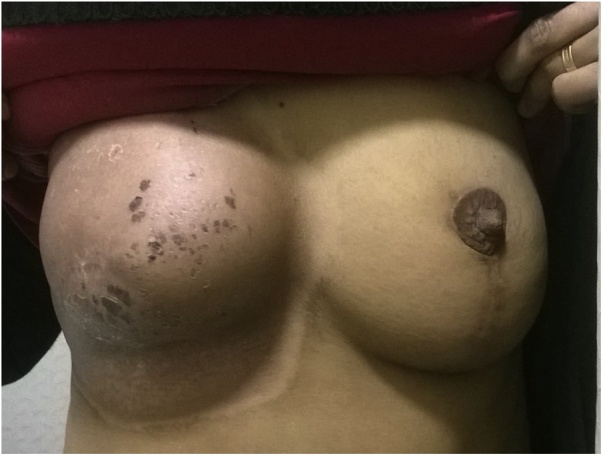
Post irradiation.

The patient was reviewed by the operating team 3 and 6 months after completion of radiation therapy regimen. An ultrasonographic examination was also performed on the reconstructed breast in each visit. There were no complications related to the implant or fat graft, only skin discoloration and some skin desquamation were noticed for which topical moisturizing creams were prescribed (Fig. 7).

## 3 Discussion

Implant based reconstruction is one of the most widely practiced methods of breast reconstruction. Implant exposure is a very devastating complication. There has been no uniform consensus on how to manage this complication in literature. Different methods have been described for salvage such as antibiotic irrigation, implant replacement and flap coverage [7].

Lipofilling is one of the new tools added to the disposal of reconstructive surgeons. The indications of this technique are still being explored in many aspects of breast reconstruction. This case report demonstrates a possible salvage and protective value for lipofilling in implant based reconstruction especially in cases where adjuvant irradiation is anticipated. In cases of implant exposure without infection fat grafting can provide a protecting layer of fat around the prosthesis.

In order to reduce the risk of implant related complications soft tissue coverage of the implant should be adequate. In patients where this soft tissue coverage is either deficient or not approved by the patient, lipofilling can play a role as an adjunct procedure that provides additional support to implant coverage.

In large series studies of lipofilling the most common complication was liponecrosis with an incidence not exceeding 2% [2]. However this complication did not occur in the displayed case.

The ultrasound guided approach was mandatory in this case in order to avoid implant puncture or pneumothorax. It proved to enhance precision and provide a more goal oriented fat injection. In addition, it allowed good estimation of the thickness and homogeneity of the fat layer injected over the target area.

Finally, it should be noted that further prospective randomized research is warranted to assess the efficacy and establish the role of lipofilling in implant based reconstruction.

## 4 Conclusion

Lipofilling is very promising as an adjunct procedure to implant based reconstruction. It can be used for implant salvage in cases of implant exposure without infection.

## Conflicts of interest

No conflicts of interest to disclose.

## Sources of funding

No funding was received to complete this work.

## Ethical approval

Approval of the ethical committee of the National Cancer Institute of Cairo University was obtained to publish this data.

## Consent

An informed consent for publication of personal and medical data was obtained from the patient.

## Author’s contribution

All authors contributed evenly to the publication of this case report.

## Registration of research studies

N/A.

## Guarantor

Dr Tarek Hashem.

## Provenance and peer review

Not commissioned, externally peer-reviewed.
